# Comparison between Sampling Techniques for Virological Molecular Analyses: Dolphin Morbillivirus and Herpesvirus Detection from FTA^®^ Card and Frozen Tissue

**DOI:** 10.3390/v15122422

**Published:** 2023-12-13

**Authors:** Haiyang Si, Claudia Maria Tucciarone, Mattia Cecchinato, Matteo Legnardi, Sandro Mazzariol, Cinzia Centelleghe

**Affiliations:** 1 Department of Animal Medicine, Production and Health (MAPS), University of Padua, Viale dell’Università 16, 35020 Legnaro, Italy; haiyang.si@phd.unipd.it (H.S.); mattia.cecchinato@unipd.it (M.C.); matteo.legnardi@unipd.it (M.L.); 2 Department of Comparative Biomedicine and Food Science (BCA), University of Padua, Viale dell’Università 16, 35020 Legnaro, Italy; sandro.mazzariol@unipd.it (S.M.); cinzia.centelleghe@unipd.it (C.C.)

**Keywords:** molecular detection, FTA^®^ card imprints, frozen tissue, dolphin morbillivirus, herpesvirus

## Abstract

Stranded animals offer valuable information on marine mammal physiology and pathology; however, the decomposition state of the carcasses and lack of a rigorous cold chain for sample preservation can sometimes discourage diagnostic analyses based on nucleic acid detection. The present paper aims at evaluating the reliability of FTA^®^ card tissue imprints as an alternative matrix to frozen tissues for virological analyses based on biomolecular methods. Given the contribution of Cetacean morbillivirus (CeMV) to strandings and the increase of herpesvirus detection in cetaceans, these two pathogens were selected as representative of RNA and DNA viruses. Dolphin morbillivirus (DMV) and herpesvirus presence was investigated in parallel on tissue imprints on FTA^®^ cards and frozen tissues collected during necropsy of dolphins stranded in Italy. Samples were analysed by nested RT-PCR for DMV and nested-PCR for herpesvirus. Only one animal was positive for herpesvirus, hampering further considerations on this virus. DMV was detected in all animals, both in FTA^®^ card imprints and tissue samples, with differences possibly related to the decomposition condition category of the carcasses. Tissue sampling on FTA^®^ cards seems a promising alternative to frozen tissues for biomolecular analyses, especially when ensuring adequate storage and shipment conditions for frozen tissues is difficult.

## 1. Introduction

The study of marine mammal health status can be difficult and partial, as for wildlife in general, since planning and performing an active surveillance in this field can be highly interfering and impactful on animal welfare [1]. Capture and release of cetaceans for sampling purposes are deeply invasive procedures and require highly specialized teams and logistics, in addition to special permits [2]. An alternative to capture can be blubber sampling of live free-ranging animals through dart biopsy, which is less invasive, but it allows only for a narrow range of analyses [3]. Complex new techniques for the collection of samples from live animal, such as blow [4,5,6] or excrement [7,8] collection by drones, have been developed to gather information by non-invasive means, even though they require large efforts from the research teams.

Because of the current great interest in marine mammals, many challenges to collection methods still need to be overcome. Appropriate collection methods provide access to large amounts of information, both on physiological and pathological aspects of marine mammals. Nevertheless, passive surveillance based on stranded animals can be limiting. When dealing with dead stranded animals or animals dying ashore, other factors can affect the quality of the sampling, such as the decomposition state of the carcass, weather conditions, accessibility to the stranding site, logistics, technical skills and resources, specimen storage options, and the requirements of the different diagnostic tests [9].

Pathological examinations are usually performed on formalin-fixed tissues, which can be conserved at room temperature once soaked in formalin, whereas tissues for microbiological and virological analyses require controlled cold temperatures for storage and shipment. Moreover, the avoidance of nucleic acid degradation or microbial proliferation is an additional constraint, thus the quality of such samples can hardly be preserved from the first steps on the field [9,10,11]. Nucleic acid-stabilizing solutions can be helpful especially to preserve RNA, allowing at least a freezing delay, although pathogens might remain infectious within the sample [12]. Nonetheless, extreme field conditions, the lack of resources guaranteeing the cold chain, or the advanced decomposition state of the carcass can a priori discourage sampling for virological analyses, whose results can be occasionally affected by nucleic acid degradation and inhibiting substances [13]. As a consequence, the loss of important information about factors contributing to death and stranding, such as viral or bacterial infections [14,15,16], impoverishes the case description and limits the understanding of epidemiology and the population health status assessment. In ideal conditions, a multidisciplinary approach should always be adopted during necropsies and case studies, with the important contribution of microbiological and molecular analyses, that should not be neglected.

In fact, infections are common findings in cetaceans and their role in animal death or strandings has been discussed both as a primary cause and secondary to other conditions, such as immunosuppression due to environmental pollutants, reduced fishing resources, and other anthropogenic factors [17,18,19,20]. Of particular relevance, Cetacean morbillivirus (CeMV), along with other microorganisms such as *Brucella ceti* and *Toxoplasma gondii* [17] are recognized as causes of notable outbreaks worldwide, especially along the Atlantic and Mediterranean coastlines [20]. 

In particular, CeMV causes respiratory and neurological disease, that can impair swimming and diving, and it also promotes immunosuppression by lymphoid depletion, favoring in turn secondary infections [18]. CeMV possesses a certain genetic heterogeneity that resulted in the recognition of different strains, which have been tentatively proposed as subspecies (CeMV-1/5) [20,21]. Each strain was firstly identified in precise hosts, but they are likely exchanged among various marine mammal species [20]. Dolphin morbillivirus (DMV, CeMV-1) was firstly detected in Mediterranean striped dolphins (*Stenella coeruleoalba*), porpoise morbillivirus (PMV, CeMV-2) in harbor porpoises (*Phocoena phocoena*), and pilot whale morbillivirus (PWMV, CeMV-4) in a long-finned pilot whale (*Globicephala melas*) [18]. Three new but less characterized strains were detected in a Longman’s beaked whale (*Indopacetus pacificus*) (CeMV-3), in a Guiana dolphin (*Sotalia guianensis*) (CeMV-5), and in Indo-Pacific bottlenose dolphins (*Tursiops aduncus*) [18]. 

The scarcity of detections narrows the number of available genomes for the comparison of new data, and the low quality of samples often prevents the collection of adequate amounts of genetic information to perform more in-depth studies. In fact, new high-throughput sequencing tools paired with bioinformatic analyses, such as phylogenetic studies, could shed light on the origin, spread, and evolution of CeMV [20]. Not so many studies are available [20,21,22,23] and involve few genetic sequences, but they reveal ample margins of discoveries. CeMV evolutionary patterns have involved host jumps [24] and could further produce the emergence of new strains, as it has been already confirmed for other morbilliviruses [25]. 

The severity of the disease, CeMV geographic distribution, the susceptibility of cetacean and other marine mammal species should prompt a systematic investigation towards this pathogen, that should not be dismissed due to the sample unsuitability. In fact, it is still not clear whether the lack of information from specific geographical areas (i.e., Indian and Pacific Oceans) is due to local features of viral circulation or to the abovementioned logistic limitations, reducing the capacity of the stranding networks to promptly intervene and reach a definitive assessment of the causes of death or stranding, including infectious agents.

Similarly, a great interest is rising with regard to herpesviruses, which have been identified in cetaceans [26,27,28,29,30,31,32,33], often in coinfection with CeMV, whose immunosuppressive effect could favor herpesvirus entrance [30]. Alpha and gammaherpesvirus have been detected in relation with systemic or localized forms [27,32,33,34,35,36], and their pathogenetic role has not been completely outlined yet. Herpesviruses are considered to maintain their characteristic ability to remain latent also in cetaceans, likely causing problems also after reactivation [37] and further complicating their individuation. Due to their heterogeneity and incomplete characterization [37], molecular methods often rely on generic assays for herpesvirus detection [38], allowing a broader identification possibly to the detriment of a higher sensitivity, that could be achieved by more targeted assays. 

In addition to specific distribution patterns or intrinsic resistance features of each pathogen, other important constraints are linked to sampling techniques, the quality of the starting samples, the decomposition state of the carcasses, logistics, and resources, especially in this field.

For this reason, Whatman FTA^®^ cards were considered as a possible alternative to frozen tissue sampling for biomolecular analyses. Whatman FTA^®^ cards are commercial products consisting of filter paper soaked in a chemical mix which lyses cells, prevents growth of bacteria, and protects nucleic acids in the samples [39]. FTA^®^ card imprints are already widely adopted in veterinary microbiology [40,41,42,43], because of many advantages, such as room temperature storage, the lack of biohazard of the specimen, minimal contamination risks, long storage period and pocket-sized volume, easy shipment, and transferal to laboratories [42,43,44,45]. 

This study aimed at a preliminary evaluation of the utility of FTA^®^ card sampling in comparison to frozen tissue sampling, when dealing with deceased marine mammals and applied to the molecular detection of important DNA and RNA viral pathogens.

## 2. Materials and Methods

Seven dolphins were included in the study: five individuals (2 females, 3 males) belonged to the species *Stenella coeruleoalba* and two individuals (both females) belonged to the species *Tursiops truncatus*. All animals were found stranded in different locations along the Italian coastlines in the period 2022–2023 (Table 1) and were classified following the decomposition condition categories (DCC) proposed by the European best practice on cetacean post-mortem investigation and tissue sampling document [9].

At the time of retrieval after stranding, two *Stenella coeruleoalba* and one *Tursiops truncatus* were scored as DCC 2, whereas the remaining four animals were classified as DCC 3 (Table 1). Animals were transferred to the Department of Comparative Biomedicine and Food Science (BCA) in Legnaro, Italy, where the carcasses were frozen at −20 °C, until necropsy.

The carcasses were thawed at room temperature for 24 h before necropsy. Samples were collected during routine post-mortem examination by personnel of the Department of Comparative Biomedicine and Food Science (BCA), following the standard protocol described by the European best practice on cetacean post-mortem investigation and tissue sampling document [9]. During the necropsy, organs were extracted, and tissue aliquots were collected for molecular analyses based on viral tissue tropism. Tissues were imprinted on FTA^®^ cards (QIAcard FTA^®^ Classic, Qiagen, Hilden, Germany) by smearing a cut surface of around 1 cm^2^ of tissue on each FTA^®^ card circle for 5 s, then FTA^®^ cards were air dried before being sealed in separate plastic bags for each individual. FTA^®^ cards were stored at room temperature (~24–26 °C) for around 10 days (1–2 weeks) until processing. Tissue aliquots from the same imprinted region were stored in parallel in 2 mL tubes at −80 °C until processing.

FTA^®^ card and frozen tissue samples were processed together for each individual. Tissues were sampled, when possible, based on the decomposition state of the carcass. In detail, aliquots and imprints were collected from brain, cerebellum, kidney, liver, lung, prescapular and pulmonary lymph nodes, spleen, and tonsils. Samples were transferred to the Department of Animal Medicine Production and Health (MAPS) in Legnaro, Italy, for molecular analyses.

For RNA extraction, samples were initially prepared following two different procedures for tissues and FTA^®^ cards. Then, 25 or 30 mg of tissue were transferred to a 2 mL screw cap tube with a stainless bead and homogenized with RLT buffer provided in RNeasy Plus Mini kit (Qiagen, Hilden, Germany) using TissueLyser II instrument (Qiagen, Hilden, Germany). A 5 mm^2^ piece of FTA^®^ card was cut and incubated in RLT buffer in a shaker at 1500 rpm for 1 h at room temperature. Samples were then processed with RNeasy Plus Mini kit (Qiagen, Hilden, Germany) with a common protocol, following the manufacturer’s instructions.

For DNA extraction, samples were prepared following two different approaches for tissues and FTA^®^ cards. Around 25 mg of tissue were transferred to a 2 mL screw cap tube with a stainless bead and homogenized with ATL buffer provided in Dneasy Blood & Tissue kit (Qiagen, Hilden, Germany) using TissueLyser II instrument (Qiagen, Hilden, Germany). A 5 mm^2^ piece of FTA^®^ card was cut and incubated in 280 µL of ATL buffer in a shaker at 1500 rpm for 12 h at 65 °C, as suggested by the literature [46]. Samples were then processed with Dneasy Blood & Tissue kit (Qiagen, Hilden, Germany) with a common protocol, following the manufacturer’s instructions. Extracted RNA samples were tested using a previously validated nested RT-PCR for the detection of DMV [47]. RT-PCR assay was performed using SuperScript III One-Step RT-PCR System with Platinum Taq DNA Polymerase kit (Thermofisher Scientific, Waltham, MA, USA) for the first amplification round, then Platinum™ II Taq Hot-Start DNA Polymerase kit (Thermofisher Scientific, Waltham, MA, USA) was used for the second amplification. Both rounds of amplification were run on Applied Biosystems 2720 Thermal Cycler (Thermofisher Scientific, Waltham, MA, USA). Extracted DNA samples were tested using a previously validated nested PCR for pan-herpesvirus detection [38]. Nested-PCR assay was performed using Platinum™ II Taq Hot-Start DNA Polymerase kit (Thermofisher Scientific, Waltham, MA, USA) for both amplifications, which were also run on Applied Biosystems 2720 Thermal Cycler (Thermofisher Scientific, Waltham, MA, USA).

PCR results were evaluated by agarose gel electrophoresis, the lack of contamination was assured by a negative control included in all runs, and amplicon specificity was assessed by comparison with a positive control (viral isolate for DMV, feline herpesvirus vaccine for herpesvirus) included in all runs.

To compare the sensitivity of the two sampling methods, organs were considered positive when viruses were amplified from at least one of the two respective matrices, and the differences in detection rate between tissues and FTA^®^ cards were assessed with the two-tailed Fisher’s exact test [48]. The analysis was performed using the stats package included in R [49], setting the significance level at *p* < 0.05.

Extracted RNA and DNA samples were tested using SYBR-based real time RT-PCR and PCR assays, slightly modified from Spinsanti et al. [50], targeting transcript and genomic sequences of a housekeeping gene (glyceraldehyde-3P-dehydrogenase, GAPDH) as internal control. The assays were performed using SuperScript™ III Platinum™ SYBR™ Green One-Step qRT-PCR Kit (Thermofisher Scientific, Waltham, MA, USA) and PowerUp™ SYBR™ Green Master Mix (Applied Biosystems™, Waltham, MA, USA) on LightCycler^®^ 96 Instrument (Roche, Basil, Switzerland) to evaluate the presence of nucleic acids in the samples after the extraction.

## 3. Results

A total of 53 tissue aliquots and respective imprints were collected, consisting of 106 samples. Depending on the decomposition state of the carcasses and the conservation of the sampled organs, samples were collected from brain, kidney, liver, lung, and spleen of all individuals (7/7); prescapular and pulmonary lymph nodes were collected from five out of seven individuals; cerebellum and tonsils were collected from four out of seven individuals (Table 2). 

All RNA and DNA extracts were positive for the genomic internal control, confirming the extraction of nucleic acids (RNA Cq values: min 13.53; max 29.83; mean 22.06; median 22.11; DNA Cq values min 15.9; max 30.11; mean 21.17; median 20.79). Regarding viral identification, only one animal (ID 616, coded DCC 3) was positive for herpesvirus (Table 2): viral DNA was detected both from FTA^®^ card imprint and tissue of the pulmonary lymph node, and only from the FTA^®^ card imprint of the spleen.

Eighteen (18/53, 34%) tissue samples and twenty-eight (28/53, 53%) FTA^®^ card imprints were positive for DMV (Table 2). All animals were positive for DMV, which was detected both in tissues and FTA^®^ cards, except for animal ID 616 (Table 2): only FTA^®^ card imprints of brain and kidney were positive for this individual, which was classified as DCC 3. FTA^®^ card imprint and tissue positivity matched for more than one organ in four out of seven individuals (Table 2): individuals ID 619 and ID 622 were both coded as DCC 2, and DMV was detected both in FTA^®^ card imprint and tissue samples of kidney and prescapular lymph node; individuals ID 620 and ID 623 were both coded as DCC 3, and FTA^®^ card imprint and tissue positivity matched for cerebellum and kidney (ID 620), and brain and liver (ID 623), respectively. Finally, DMV was detected in both FTA^®^ card imprint and tissue samples of lung from individual ID 624 (DCC 2). 

When considering the target organs, the positivity of tissues and FTA^®^ card imprints was more often in accordance with kidney (three out of seven animals, ID 619, 620, 622) and pulmonary lymph node (two out of seven animals, ID 619, 622), whereas it was in accordance with one case for brain (ID 623), cerebellum (ID 620), liver (ID 623), and lung (ID 624) (Table 2). Based on DCC, at least one matching positivity was present between FTA^®^ card imprints and tissues of all DCC 2 coded individuals (ID 619, 622, 624), in comparison with only half of DCC 3 coded individuals (ID 620, 623).

Considering the 37 organs which tested positive using one or both sampling methods, a significant difference (*p* = 0.03) was found when comparing the detection rate obtained with FTA^®^ cards (28 positives, 76%) and tissues (18 positives, 49%). The difference was still significant among the 19 positive organs from DCC 3 coded individuals (*p* = 0.007), but it was non-significant among the 18 positive organs from DCC 2 coded individuals.

## 4. Discussion

The present study evaluated a sampling approach for virological molecular analyses alternative to tissue sampling, which requires cold chain maintenance, especially when working with already damaged tissues [9]. Stranded cetaceans often present with poor carcass conditions, due to weather conditions accelerating decomposition, long period in water before arriving ashore [51], exposure to scavenging animals [52], and other causes promoting post-mortem processes (i.e., size of the carcass, blubber thickness, type of tissues, gut microflora proliferation, etc.) [47,51,53] and increasing the uncertainty of the time of death assessment [51]. In light of all these factors, the stabilization of the samples has to be assured as soon as they are extracted for pathological examination and formalin fixation is routinely performed directly on the field in dedicated containers [9]. 

Freezing is also fundamental for virological investigations, to preserve nucleic acids [10,11] and increase the chances of virus detection [9]; however, it is not always possible to control the sample temperature immediately after collection, especially when performing necropsies on the field [10]. In cases where samples have been collected for histology only, nucleic acid extraction can be attempted on formalin-fixed paraffin-embedded samples, although the nucleic acid yield is often inadequate [54]. Therefore, tissue imprints on FTA^®^ cards might represent an effective and convenient alternative, allowing a rapid stabilization of the specimen and the fixation of nucleic acids on the filter paper, blocking microorganism proliferation and autolytic processes, which might affect the retrieval of target nucleic acids, particularly for delicate pathogens, such as RNA viruses [55]. Furthermore, FTA^®^ cards allow the simultaneous storage of multiple samples and lower the risk of contamination [11,56], with a cost-effective option in comparison to nucleic acid stabilizing solutions, requiring dedicated tubes and only delaying freezing [9]. On the other hand, issues related to sensitivity or viral distribution within the specimen might become relevant [47], influencing the effectiveness of the sampling. 

Regarding the detection of herpesvirus in the present study, only one animal (ID 616) was positive, indicating a likely lower circulation of herpesvirus in the Mediterranean population, if compared to DMV. This individual was coinfected with herpesvirus and DMV, as frequently encountered [30,32,33]. Concerning the aim of our study, no definitive conclusions can be inferred on this sampling approach for DNA viruses. Nonetheless, viral DNA was detected both in FTA^®^ card imprint and tissue samples of a pulmonary lymph node and FTA^®^ card imprint of the spleen, suggesting the suitability of this method. A greater DNA stability is acknowledged if compared to RNA [57], thus a certain superior ease in DNA preservation and retrieval could be anticipated, but the effects of autolytic processes and environmental conditions on the carcasses must not be disregarded. Therefore, this promising tool should be considered and further studied for the application in this field, notably in light of the already large use of FTA^®^ cards for the identification and preservation of DNA [58,59,60,61]. 

Unfortunately, neither an evaluation of viral tissue distribution nor genetic characterization was within the scope of this study, but the detection of herpesvirus reinforces the need to include this pathogen in systematic testing of stranded animals to deepen these aspects. 

Although partially expected given the reported high frequency of DMV infections [18], the positivity of all individuals for DMV was in fact a remarkable finding, especially for the particular structure and aim of this study.

Statistically significant differences were found in the detection rates achieved by relying on FTA^®^ cards and tissue samples, favoring the former, and were mostly ascribable to samples collected from DCC 3 coded animals compared to fresher ones. These results may suggest that nucleic acid fixation and conservation on FTA^®^ cards might have been beneficial when dealing with poorly conserved specimens, if compared with tissue samples that underwent a further process of freezing and thawing before RNA extraction and processing. In this case, tissues were frozen within a short time after necropsy, whereas it is not always possible to ensure the same rapidity for a correct preservation process in field conditions [10,11]. 

Another relevant finding is the inconsistency between results obtained with tissues and FTA^®^ cards, with 25 out of 37 DMV-positive organs being confirmed when processed with only one of the two methods (Table 2). Several factors could have contributed to this variability. Differences in the detection rate due to the viral titer cannot be excluded, since the assay used in this study is not quantitative [47]; however, a low viral titer could be possibly transferred less efficiently to FTA^®^ cards, also considering the need of standardization of the tissue imprinting process. Likewise, a high viral load could allow the nucleic acids to persist more easily and be detected in tissues, despite advanced decomposition affecting RNA detection [62].

Few other hypotheses can be proposed about the reasons behind the detection inconsistency (Table 2) between the two sampling approaches. The main concern in CeMV detection is linked to its multifocal distribution in many tissues, especially in the brain and lungs [18], that might be missed both by direct tissue sampling and imprinting, if lesions are not macroscopically evident, but also during the preparation of samples for extraction, which requires the use of small tissue quantities (i.e., to avoid the overload of the silica membrane of the column-based extraction kits). On the other hand, the preparation of the imprints during the sampling procedures of the organs could expose a fresher cut surface of the tissue to the filter paper, collecting fluids and cell material more abundant in virus, which could further deteriorate during the following freezing/thawing processes of the tissue samples [47]. A particular contribution of decomposition might be assumed in this case, given the sample nature, even though other studies evidenced an equal performance of FTA^®^ cards compared to frozen matrices [44,63]. 

Conversely, for firm tissues like the spleen, homogenization could be a problematic process, especially after freezing, thawing, and fluid loss. The complete lysis of the dehydrated tissue might be difficult to achieve and, at the same time, the abundance of the residual tissue might interfere with the silica membrane of the column, limiting the nucleic acid yield and the extraction efficiency. In addition to a possible lower preservation of the tissue [47], this could possibly explain the lower viral detection rate in spleen tissue samples rather than in spleen FTA^®^ card imprints.

## 5. Conclusions

Marine mammal mortalities are thoroughly investigated along the European, North American, and Oceania coastlines, where fatalities are constantly monitored by stranding networks. Since there is an increasing interest in exploring cetacean strandings in poorly monitored waters (i.e., in Indian and Pacific Oceans) and in regions with logistic difficulties, validated methodologies for sampling and preserving essential tissues for ancillary examination should be explored, especially if they allow the avoidance of cold technologies.

In the present study, both classical and alternative matrices consisting of tissues imprinted on FTA^®^ cards allowed the detection of DNA and RNA viruses. Notably, DMV was detected at least in one FTA^®^ card sample for each analyzed organ, suggesting the suitability of this sampling approach for a range of different specimens. In addition, the detection rate achieved with FTA^®^ cards appeared higher than with tissues, especially when dealing with less conserved specimens, suggesting that such an approach might even be advantageous for molecular biology investigation in some contexts.

Alternative sampling strategies, such as FTA^®^ cards, might also present some practical advantages, compared to tissue sampling, which is not always feasible in field conditions during marine mammals stranding events and mortalities, and might not assure a good quality of the sample, if not stored correctly. For instance, FTA^®^ cards allow a microbiologically safe and easy storage that does not require cold shipping or particular biohazard precautions, since the biological substance is chemically inactivated on the paper surface.

Further studies are required, but encouraging results are provided and this sampling procedure can be positively embraced, especially for cases where tissue sampling cannot be performed because of limited resources for storage or shipment, but microbiological and/or virological molecular analyses are considered pivotal.

## Figures and Tables

**Table 1:** Summary of the information related to the animals considered in the study, Italian provinces where the stranding sites were located are indicated between parentheses. F: female; M: male; DCC: decomposition condition category.

ID	Species	Sex	Length (cm)	Weight (kg)	DCC	Standing Site	Stranding Date
616	*Tursiops truncatus*	F	170	59.5	3	Lido delle Nazioni (FE)	21 October 2022
619	*Stenella coeruleoalba*	F	195	61	2	Orbetello (GR)	21 August 2022
620	*Stenella coeruleoalba*	M	171	52	3	Lecce (LE)	24 March 2022
621	*Stenella coeruleoalba*	F	80	6.45	3	Maruggio (TA)	19 August 2022
622	*Stenella coeruleoalba*	M	91	7.1	2	Vittoria (RG)	13 July 2022
623	*Stenella coeruleoalba*	M	90	7.45	3	Messina (ME)	4 August 2022
624	*Tursiops truncatus*	F	185	80.5	2	Porto Tolle (RO)	25 February 2023

**Table 2:** DMV detection results from FTA^®^ card imprint and tissue comparison. 1 = positive, 0 = negative, “ns” non sampled organs. DCC: decomposition condition category.

ID	616		619		620		621		622		623		624	
DCC	3		2		3		3		2		3		2	
Organ	Imprint	Tissue	Imprint	Tissue	Imprint	Tissue	Imprint	Tissue	Imprint	Tissue	Imprint	Tissue	Imprint	Tissue
Brain	1	0	0	0	0	0	0	1	1	0	1	1	0	1
Cerebellum	ns	ns	1	0	1	1	ns	ns	1	0	1	0	ns	ns
Kidney	1	0	1	1	1	1	1	0	1	1	1	0	0	0
Liver	0	0	0	1	0	0	1	0	0	0	1	1	1	0
Lung	0	0	0	1	1	0	0	1	0	0	0	0	1	1
Prescapular lymph node	ns	ns	ns	ns	0	1	1	0	1	0	1	0	0	0
Pulmonary lymph node	0 *	0 *	1	1	1	0	ns	ns	1	1	ns	ns	0	1
Spleen	0 *	0	1	0	1	0	0	0	1	0	0	0	0	1
Tonsils	ns	ns	ns	ns	ns	ns	1	0	0	1	0	0	0	0
Total of positive samples	2	0	4	4	5	3	4	2	6	3	5	2	2	4

* Positive samples for herpesvirus.

## Data Availability

Data are contained within the article.
